# Supplementary material for: Association of lymphopenia and RDW elevation with risk of mortality in acute aortic dissection
Source: PLoS One. 2023 Mar 15;18(3):e0283008. doi: 10.1371/journal.pone.0283008 (PMC10016706; doi:10.1371/journal.pone.0283008)
Supplement: S1 Table — (DOCX) [file pone.0283008.s002.docx]

RESEARCH ARTICLE

**Association of Lymphopenia and RDW Elevation with Risk of Mortality in Acute Aortic Dissection**

Dan Yu^1,2,3^, Peng Chen^1^, Xueyan Zhang^4^, Hongjie Wang^1,2^, Menaka Dhuromsingh^1,2^, Jinxiu Wu^6^, Bingyu Qin^4^*, Suping Guo^3,5^*, Baoquan Zhang^6^*, Chunwen Li^7^*, Hesong Zeng^1,2^*

^1^Division of Cardiology, Department of Internal Medicine, Tongji Hospital, Tongji Medical College, Huazhong University of Science and Technology, Wuhan, 430030, China

^2^Hubei Provincial Engineering Research Center of Vascular Interventional Therapy, Wuhan, 430030, China

^3^Department of Cardiac Intensive Care Unit, People’s Hospital of Zhengzhou University (Henan Provincial People's Hospital), Zhengzhou, 450003, China

^4^Department of Critical Care Medicine, Henan Key Laboratory for Critical Care Medicine, People’s Hospital of Zhengzhou University (Henan Provincial People's Hospital), Zhengzhou, 450003, China

^5^Department of Cardiac Intensive Care Unit, Central China Fuwai Hospital of Zhengzhou University (Fuwai Central China Cardiovascular Hospital), Zhengzhou, 450046, China

^6^Department of Critical Care Medicine, The Third Affiliated Hospital of Xinxiang Medical University, Xinxiang, 453000, China

^7^Department of Emergency Medicine, The Second Affiliated Hospital of Chongqing Medical University, Chongqing, 400010, China

***** Corresponding author
nicolasby@126.com (BYQ); gsp389@126.com (SPG); Zhang pzbaoq@163.com (BQZ); chunwenli@cqmu.edu.cn (CWL); zenghs@tjh.tjmu.edu.cn (HSZ)

**S1 Table. Patient Baseline Characteristics (continued)**

| **Variables** | **Total patients** | **In-hospital alive** | **In-hospital dead** | ***P*-value** |
| --- | --- | --- | --- | --- |
| N | 1903 | 1430 | 473 |  |
| Heart rate, median (IQR), bpm | 80.0 (70.0-90.0) | 80.0 (70.0-90.0) | 80.0 (69.0-90.0) | 0.56 |
| Systolic blood pressure, median (IQR), mm Hg | 137.0 (121.0-155.0) | 139.0 (123.2-158.0) | 128.0 (108.0-147.0) | <0.001 |
| Diastolic blood pressure, median (IQR), mm Hg | 77.0 (66.0-89.0) | 79.0 (68.0-90.0) | 71.0 (60.0-85.0) | <0.001 |
| Respiratory rate, median (IQR), bpm | 20.0 (18.0-20.0) | 20.0 (18.0-20.0) | 20.0 (19.0-20.0) | 0.194 |
| Temperature, median (IQR), ℃ | 36.5 (36.3-36.8) | 36.5 (36.3-36.8) | 36.5 (36.3-36.8) | 0.259 |
| White blood cell count, median (IQR), ×10^9^/L | 11.4 (8.6-14.5) | 11.1 (8.2-14.0) | 12.6 (9.7-16.2) | <0.001 |
| Absolute neutrophil count, median (IQR), ×10^9^/L | 9.7 (6.8-12.6) | 9.3 (6.5-12.1) | 10.9 (7.9-14.2) | <0.001 |
| Neutrophil percentage, median (IQR), % | 84.8 (77.7-89.3) | 84.1 (76.1-88.8) | 86.8 (81.8-90.3) | <0.001 |
| Absolute monocyte count, median (IQR), ×10^9^/L | 0.7 (0.4-0.9) | 0.6 (0.4-0.9) | 0.7 (0.5-1.0) | <0.001 |
| Monocyte percentage, median (IQR), % | 6.1 (4.3-8.2) | 6.2 (4.3-8.3) | 5.9 (4.1-7.9) | 0.086 |
| Absolute eosinophil count, median (IQR), ×10^9^/L | 0.01 (0.00-0.04) | 0.01 (0.00-0.05) | 0.01 (0.00-0.02) | <0.001 |
| Eosinophil percentage, median (IQR), % | 0.10 (0.00-0.40) | 0.10 (0.00-0.50) | 0.00 (0.00-0.20) | <0.001 |
| Absolute basophil count, median (IQR), ×10^9^/L | 0.01 (0.01-0.02) | 0.01 (0.01-0.03) | 0.01 (0.01-0.02) | 0.047 |
| Basophil percentage, median (IQR), % | 0.10 (0.10-0.20) | 0.10 (0.10-0.24) | 0.10 (0.10-0.20) | <0.001 |
| Absolute lymphocyte count, median (IQR), ×10^9^/L | 0.98 (0.68-1.36) | 1.00 (0.69-1.41) | 0.91 (0.63-1.24) | <0.001 |
| Red blood cell count, median (IQR), ×10^12^/L | 4.3 (3.8-4.7) | 4.3 (3.9-4.7) | 4.2 (3.8-4.7) | 0.063 |
| Hemoglobin, median (IQR), g/L | 131.0 (116.0-142.0) | 132.0 (117.0-143.0) | 128.0 (115.0-140.0) | 0.039 |
| Hematocrit, median (IQR), % | 38.8 (34.8-41.9) | 39.0 (35.0-42.0) | 38.0 (34.5-41.8) | 0.077 |
| MCV, median (IQR), fL | 90.7 (87.7-93.9) | 90.6 (87.7-93.8) | 90.9 (87.8-94.1) | 0.41 |
| MCH, median (IQR), pg | 30.6 (29.5-31.6) | 30.6 (29.5-31.6) | 30.6 (29.4-31.7) | 0.91 |
| MCHC, median (IQR), g/L | 336.0 (328.0-344.0) | 337.0 (328.0-344.0) | 335.0 (327.0-344.0) | 0.096 |
| RDW-CV, median (IQR), % | 13.2 (12.7-13.9) | 13.2 (12.6-13.9) | 13.3 (12.9-13.9) | <0.001 |
| Platelet count, median (IQR), ×10^9^/L | 160.0 (125.0-198.0) | 161.0 (129.0-203.0) | 153.0 (120.0-189.0) | <0.001 |
| PDW, median (IQR), fL | 12.8 (11.3-15.3) | 12.8 (11.2-15.4) | 12.7 (11.3-14.9) | 0.471 |
| MPV, median (IQR), fL | 10.5 (9.7-11.3) | 10.4 (9.7-11.3) | 10.6 (9.7-11.5) | 0.098 |
| Plateletcrit, median (IQR), % | 0.17 (0.14-0.21) | 0.17 (0.14-0.21) | 0.16 (0.13-0.20) | <0.001 |
| P-LCR, median (IQR), % | 29.3 (23.2-36.2) | 29.0 (23.2-35.8) | 29.8 (23.2-37.0) | 0.277 |
| APTT, median (IQR), seconds | 37.3 (33.7-41.3) | 36.9 (33.2-40.5) | 38.5 (34.8-43.6) | <0.001 |
| TT, median (IQR), seconds | 16.7 (15.6-18.0) | 16.5 (15.5-17.7) | 17.3 (16.0-19.1) | <0.001 |
| PT, median (IQR), seconds | 14.2 (13.3-15.2) | 14.1 (13.1-14.9) | 14.8 (14.0-15.9) | <0.001 |
| INR, median (IQR) | 1.1 (1.0-1.2) | 1.1 (1.0-1.2) | 1.2 (1.1-1.3) | <0.001 |
| PTA, median (IQR), % | 83.0 (74.0-93.0) | 85.0 (75.9-95.0) | 77.0 (68.0-86.0) | <0.001 |
| Fibrinogen, median (IQR), g/L | 2.8 (2.1-3.8) | 2.9 (2.2-4.0) | 2.4 (1.8-3.3) | <0.001 |
| ALT, median (IQR), U/L | 19.0 (13.0-33.0) | 19.0 (13.0-31.0) | 22.0 (13.0-41.2) | <0.001 |
| AST, median (IQR), U/L | 22.0 (16.0-33.0) | 21.0 (16.0-30.0) | 26.0 (18.0-51.5) | <0.001 |
| Albumin, median (IQR), g/L | 39.0 (36.0-41.8) | 39.4 (36.3-42.0) | 38.0 (35.3-41.0) | <0.001 |
| Globulin, median (IQR), g/L | 26.2 (23.3-29.3) | 26.4 (23.5-29.7) | 25.6 (22.8-28.3) | <0.001 |
| Total bilirubin, median (IQR), µmol/L | 14.2 (10.3-19.8) | 14.0 (10.2-19.6) | 14.5 (10.4-19.9) | 0.198 |
| Direct bilirubin, median (IQR), µmol/L | 4.7 (3.4-6.8) | 4.7 (3.4-6.6) | 4.9 (3.5-7.3) | 0.11 |
| Indirect bilirubin, median (IQR), µmol/L | 9.2 (6.3-12.9) | 9.1 (6.3-12.9) | 9.3 (6.8-12.8) | 0.297 |
| Uric acid, median (IQR), µmol/L | 353.0 (278.0-439.0) | 340.1 (271.8-422.0) | 385.9 (308.7-472.2) | <0.001 |
| Urea nitrogen, median (IQR), mmol/L | 6.5 (5.1-8.3) | 6.3 (4.9-7.9) | 7.6 (6.0-9.7) | <0.001 |
| Creatinine, median (IQR), µmol/L | 85.0 (67.0-115.0) | 81.0 (65.0-107.0) | 102.0 (76.0-143.5) | <0.001 |
| Serum glucose, median (IQR), mmol/L | 6.9 (5.9-8.1) | 6.8 (5.8-7.9) | 7.5 (6.3-9.0) | <0.001 |
| Sodium, median (IQR), mmol/L | 139.5 (137.4-141.5) | 139.4 (137.3-141.4) | 139.7 (137.6-141.8) | 0.096 |
| Potassium, median (IQR), mmol/L | 4.1 (3.7-4.4) | 4.0 (3.7-4.3) | 4.2 (3.8-4.6) | <0.001 |
| Chlorine, median (IQR), mmol/L | 102.4 (100.0-104.8) | 102.2 (99.9-104.5) | 102.8 (100.8-105.1) | <0.001 |
| Calcium, median (IQR), mmol/L | 2.2 (2.1-2.2) | 2.2 (2.1-2.3) | 2.1 (2.1-2.2) | <0.001 |

Continuous variables are represented as median (IQR) and categorical variables as numbers (%).

NA, not applicable; IQR, interquartile range; MCV, mean corpuscular volume; MCH, mean corpuscular hemoglobin; MCHC, mean corpuscular hemoglobin concentration; RDW-CV, red cell volume distribution width-CV; PDW, platelet distribution width; MPV, mean platelet volume; P-LCR, platelet-large cell rate; APTT, activated partial thromboplastin time; TT, thrombin time; PT, prothrombin time; PTA, prothrombin time activity; INR, international normalized ratio; ALT, alanine transaminase; AST, aspartate transaminase;
