# Supplementary material for: Association of lymphopenia and RDW elevation with risk of mortality in acute aortic dissection
Source: PLoS One. 2023 Mar 15;18(3):e0283008. doi: 10.1371/journal.pone.0283008 (PMC10016706; doi:10.1371/journal.pone.0283008)
Supplement: S2 Table — (DOCX) [file pone.0283008.s003.docx]

RESEARCH ARTICLE

**Association of Lymphopenia and RDW Elevation with Risk of Mortality in Acute Aortic Dissection**

Dan Yu^1,2,3^, Peng Chen^1^, Xueyan Zhang^4^, Hongjie Wang^1,2^, Menaka Dhuromsingh^1,2^, Jinxiu Wu^6^, Bingyu Qin^4^*, Suping Guo^3,5^*, Baoquan Zhang^6^*, Chunwen Li^7^*, Hesong Zeng^1,2^*

^1^Division of Cardiology, Department of Internal Medicine, Tongji Hospital, Tongji Medical College, Huazhong University of Science and Technology, Wuhan, 430030, China

^2^Hubei Provincial Engineering Research Center of Vascular Interventional Therapy, Wuhan, 430030, China

^3^Department of Cardiac Intensive Care Unit, People’s Hospital of Zhengzhou University (Henan Provincial People's Hospital), Zhengzhou, 450003, China

^4^Department of Critical Care Medicine, Henan Key Laboratory for Critical Care Medicine, People’s Hospital of Zhengzhou University (Henan Provincial People's Hospital), Zhengzhou, 450003, China

^5^Department of Cardiac Intensive Care Unit, Central China Fuwai Hospital of Zhengzhou University (Fuwai Central China Cardiovascular Hospital), Zhengzhou, 450046, China

^6^Department of Critical Care Medicine, The Third Affiliated Hospital of Xinxiang Medical University, Xinxiang, 453000, China

^7^Department of Emergency Medicine, The Second Affiliated Hospital of Chongqing Medical University, Chongqing, 400010, China

***** Corresponding author
nicolasby@126.com (BYQ); gsp389@126.com (SPG); Zhang pzbaoq@163.com (BQZ); chunwenli@cqmu.edu.cn (CWL); zenghs@tjh.tjmu.edu.cn (HSZ)

**S2 Table. Stratified Analyses of the Associations of Lymphocyte Percentage with In-Hospital Mortality**

| **Variables** | **Deaths/N** | **Per SD increment** | ***P* value for interaction** |
| --- | --- | --- | --- |
|  |  |  |  |
| Sex | | |  |
| Female | 109/416 | 0.77 (0.60, 0.98) | 0.699 |
| Male | 364/1487 | 0.72 (0.62, 0.85) |  |
| Age, years | | |  |
| < 60 | 311/1310 | 0.73 (0.62, 0.86) | 0.745 |
| ≥ 60 | 162/593 | 0.70 (0.56, 0.88) |  |
| Anatomical classification | | |  |
| DeBakey Ⅰ | 383/1021 | 0.79 (0.68, 0.93) | 0.346 |
| DeBakey Ⅱ | 25/125 | 0.65 (0.39, 1.09) |  |
| DeBakey Ⅲa | 2/59 | 0.25 (0.02, 2.73) |  |
| DeBakey Ⅲb | 57/606 | 0.60 (0.42, 0.85) |  |
| Isolated abdominal AAD | 6/92 | 0.53 (0.24, 1.20) |  |
| Smoking history | | |  |
| No | 329/1250 | 0.73 (0.62, 0.86) | 0.821 |
| Yes | 144/653 | 0.75 (0.60, 0.94) |  |
| Hypertension history | | |  |
| No | 198/731 | 0.67 (0.55, 0.82) | 0.177 |
| Yes | 275/1172 | 0.80 (0.67, 0.96) |  |
| Diabetes history | | |  |
| No | 463/1849 | 0.73 (0.64, 0.84) | 0.753 |
| Yes | 10/54 | 0.82 (0.41, 1.64) |  |
| Onset time | | | |
| < 24h | 316/1118 | 0.70 (0.57, 0.87) | 0.824 |
| 1-7d | 141/692 | 0.75 (0.61, 0.92) |  |
| 8-14d | 16/93 | 0.80 (0.54, 1.20) |  |
| Aorta diameter | | | |
| ≥ 5.5 cm | 450/1843 | 0.77 (0.67, 0.89) | 0.051 |
| < 5.5 cm | 23/60 | 0.47 (0.28, 0.82) |  |
| Acute kidney injury | | | |
| No | 345/1628 | 0.77 (0.66, 0.90) | 0.881 |
| Yes | 128/275 | 0.75 (0.55, 1.03) |  |
| Stroke or coma | | | |
| No | 407/1784 | 0.76 (0.66, 0.88) | 0.469 |
| Yes | 66/119 | 0.64 (0.40, 1.03) |  |
| Transfusions | | | |
| No | 287/1180 | 0.73 (0.62, 0.87) | 0.687 |
| Yes | 186/723 | 0.69 (0.55, 0.87) |  |
| Limb ischemia | | | |
| No | 406/1740 | 0.73 (0.63, 0.85) | >0.99 |
| Yes | 67/163 | 0.73 (0.51, 1.06) |  |
| Procedure of operation | | | |
| None | 257/573 | 0.71 (0.60, 0.85) | 0.019 |
| Endovascular management | 32/648 | 0.23 (0.10, 0.54) |  |
| Surgical operation | 153/469 | 0.76 (0.61, 0.95) |  |
| Surgical operation and endovascular management | 31/213 | 0.73 (0.42, 1.26) |  |
| Hospital centers | | | |
| Tongji Hospital | 397/1345 | 0.78 (0.67, 0.91) | 0.511 |
| People’s Hospital of Zhengzhou University | 41/253 | 0.57 (0.38, 0.86) |  |
| Central China Fuwai Hospital of Zhengzhou University | 16/115 | 0.71 (0.43, 1.18) |  |
| Third Affiliated Hospital of Xinxiang Medical University | 11/67 | 0.53 (0.24, 1.16) |  |
| Second Affiliated Hospital of Chongqing Medical University | 8/123 | 0.69 (0.29, 1.61) |  |
| Total cholesterol |  |  |  |
| > 4mmol/l | 170/766 | 0.79 (0.63, 0.99) | 0.605 |
| ≤ 4mmol/l | 263/936 | 0.73 (0.61, 0.87) |  |
| Low density lipoprotein |  |  |  |
| < 2.6mmol/l | 210/868 | 0.68 (0.55, 0.84) | 0.397 |
| ≥2.6mmol/l | 98/450 | 0.79 (0.59, 1.07) |  |

Data was represented as HR (95% CI) with adjusted for age, sex, smoking history, hypertension history, diabetes history, aortic valve replacement history, anatomical classification, etiology, aorta diameter, onset time and hospital centers. AAD, acute aortic dissection; MFS, Marfan syndrome; BAV, Bicuspid aortic valve.
