# Supplementary material for: Association of lymphopenia and RDW elevation with risk of mortality in acute aortic dissection
Source: PLoS One. 2023 Mar 15;18(3):e0283008. doi: 10.1371/journal.pone.0283008 (PMC10016706; doi:10.1371/journal.pone.0283008)
Supplement: S4 Table — (DOCX) [file pone.0283008.s005.docx]

RESEARCH ARTICLE

**Association of Lymphopenia and RDW Elevation with Risk of Mortality in Acute Aortic Dissection**

Dan Yu^1,2,3^, Peng Chen^1^, Xueyan Zhang^4^, Hongjie Wang^1,2^, Menaka Dhuromsingh^1,2^, Jinxiu Wu^6^, Bingyu Qin^4^*, Suping Guo^3,5^*, Baoquan Zhang^6^*, Chunwen Li^7^*, Hesong Zeng^1,2^*

^1^Division of Cardiology, Department of Internal Medicine, Tongji Hospital, Tongji Medical College, Huazhong University of Science and Technology, Wuhan, 430030, China

^2^Hubei Provincial Engineering Research Center of Vascular Interventional Therapy, Wuhan, 430030, China

^3^Department of Cardiac Intensive Care Unit, People’s Hospital of Zhengzhou University (Henan Provincial People's Hospital), Zhengzhou, 450003, China

^4^Department of Critical Care Medicine, Henan Key Laboratory for Critical Care Medicine, People’s Hospital of Zhengzhou University (Henan Provincial People's Hospital), Zhengzhou, 450003, China

^5^Department of Cardiac Intensive Care Unit, Central China Fuwai Hospital of Zhengzhou University (Fuwai Central China Cardiovascular Hospital), Zhengzhou, 450046, China

^6^Department of Critical Care Medicine, The Third Affiliated Hospital of Xinxiang Medical University, Xinxiang, 453000, China

^7^Department of Emergency Medicine, The Second Affiliated Hospital of Chongqing Medical University, Chongqing, 400010, China

***** Corresponding author
nicolasby@126.com (BYQ); gsp389@126.com (SPG); Zhang pzbaoq@163.com (BQZ); chunwenli@cqmu.edu.cn (CWL); zenghs@tjh.tjmu.edu.cn (HSZ)

| Variables | Total patients | In-hospital alive | In-hospital dead | *P*-value |
| --- | --- | --- | --- | --- |
| n | 828 | 414 | 414 |  |
| Female, n (%) | 188 (22.7) | 91 (22.0) | 97 (23.4) | 0.678 |
| Age, mean median (IQR), years | 54 (48-63) | 54 (48 -63) | 54 (47-62) | 0.863 |
| Anatomical classification |  | | | |
| DeBakey Ⅰ, n (%) | 665 (80.3) | 334 (80.7) | 331 (80.0) | 0.851 |
| DeBakey Ⅱ, n (%) | 41 ( 5.0) | 19 ( 4.6) | 22 ( 5.3) |  |
| DeBakey Ⅲa, n (%) | 1 ( 0.1) | 0 ( 0.0) | 1 ( 0.2) |  |
| DeBakey Ⅲb, n (%) | 110 (13.3) | 56 (13.5) | 54 (13.0) |  |
| Isolated abdominal AAD, n (%) | 11 ( 1.3) | 5 ( 1.2) | 6 ( 1.4) |  |
| Etiology |  | | | |
| Genetic (MFS), n (%) | 10 ( 1.2) | 5 ( 1.2) | 5 ( 1.2) | 0.977 |
| Traumatic , n (%) | 5 ( 0.6) | 2 ( 0.5) | 3 ( 0.7) |  |
| Congenital disorder (BAV), n (%) | 0 (0.0) | 0 (0.0) | 0 (0.0) |  |
| Vascular inflammation (Takayasu arteritis), n (%) | 0 (0.0) | 0 (0.0) | 0 (0.0) |  |
| Infectious disease (Syphilis), n (%) | 6 ( 0.7) | 3 ( 0.7) | 3 ( 0.7) |  |
| Sporadic, n (%) | 807 (97.5) | 404 (97.6) | 403 (97.3) |  |
| History |  | | | |
| Smoking, n (%) | 233 (28.1) | 116 (28.0) | 117 (28.3) | 1 |
| Hypertension, n (%) | 473 (57.1) | 234 (56.5) | 239 (57.7) | 0.779 |
| Diabetes, n (%) | 15 ( 1.8) | 6 ( 1.4) | 9 ( 2.2) | 0.602 |
| Aortic valve replacement, n (%) | 6 ( 0.7) | 2 ( 0.5) | 4 ( 1.0) | 0.682 |
| Onset time |  | | | |
| < 24h, n (%) | 551 (66.5) | 273 (65.9) | 278 (67.1) | 0.719 |
| 1-7d, n (%) | 252 (30.4) | 130 (31.4) | 122 (29.5) |  |
| 8-14d, n (%) | 25 ( 3.0) | 11 ( 2.7) | 14 ( 3.4) |  |
| Aorta diameter |  | | | |
| ≥ 5.5 cm, n (%) | 32 ( 3.9) | 15 ( 3.6) | 17 ( 4.1) | 0.857 |
| < 5.5 cm, n (%) | 796 (96.1) | 399 (96.4) | 397 (95.9) |  |
| Hospital centers, n (%) |  | | | |
| Tongji Hospital | 686 (82.9) | 344 (83.1) | 342 (82.6) | 0.97 |
| People’s Hospital of Zhengzhou University | 70 ( 8.5) | 33 ( 8.0) | 37 ( 8.9) |  |
| Central China Fuwai Hospital of Zhengzhou University | 31 ( 3.7) | 15 ( 3.6) | 16 ( 3.9) |  |
| Third Affiliated Hospital of Xinxiang Medical University | 23 ( 2.8) | 12 ( 2.9) | 11 ( 2.7) |  |
| Second Affiliated Hospital of Chongqing Medical University | 18 ( 2.2) | 10 ( 2.4) | 8 ( 1.9) |  |

**S4 Table. Patient Baseline Characteristics (propensity score–matched population)**

Continuous variables are represented as median (IQR) and categorical variables as numbers (%).

IQR, interquartile range; AAD, acute aortic dissection; MFS, Marfan syndrome; BAV, Bicuspid aortic valve.
