# Supplementary material for: Association of lymphopenia and RDW elevation with risk of mortality in acute aortic dissection
Source: PLoS One. 2023 Mar 15;18(3):e0283008. doi: 10.1371/journal.pone.0283008 (PMC10016706; doi:10.1371/journal.pone.0283008)
Supplement: S5 Table — (DOCX) [file pone.0283008.s006.docx]

RESEARCH ARTICLE

**Association of Lymphopenia and RDW Elevation with Risk of Mortality in Acute Aortic Dissection**

Dan Yu^1,2,3^, Peng Chen^1^, Xueyan Zhang^4^, Hongjie Wang^1,2^, Menaka Dhuromsingh^1,2^, Jinxiu Wu^6^, Bingyu Qin^4^*, Suping Guo^3,5^*, Baoquan Zhang^6^*, Chunwen Li^7^*, Hesong Zeng^1,2^*

^1^Division of Cardiology, Department of Internal Medicine, Tongji Hospital, Tongji Medical College, Huazhong University of Science and Technology, Wuhan, 430030, China

^2^Hubei Provincial Engineering Research Center of Vascular Interventional Therapy, Wuhan, 430030, China

^3^Department of Cardiac Intensive Care Unit, People’s Hospital of Zhengzhou University (Henan Provincial People's Hospital), Zhengzhou, 450003, China

^4^Department of Critical Care Medicine, Henan Key Laboratory for Critical Care Medicine, People’s Hospital of Zhengzhou University (Henan Provincial People's Hospital), Zhengzhou, 450003, China

^5^Department of Cardiac Intensive Care Unit, Central China Fuwai Hospital of Zhengzhou University (Fuwai Central China Cardiovascular Hospital), Zhengzhou, 450046, China

^6^Department of Critical Care Medicine, The Third Affiliated Hospital of Xinxiang Medical University, Xinxiang, 453000, China

^7^Department of Emergency Medicine, The Second Affiliated Hospital of Chongqing Medical University, Chongqing, 400010, China

***** Corresponding author
nicolasby@126.com (BYQ); gsp389@126.com (SPG); Zhang pzbaoq@163.com (BQZ); chunwenli@cqmu.edu.cn (CWL); zenghs@tjh.tjmu.edu.cn (HSZ)

**S5 Table.** **Associations of Lymphocyte Percentage and RDW level with Risk of In-Hospital Mortality (propensity score–matched population)**

|  | Quintiles of the exposure | | | | |  |  |
| --- | --- | --- | --- | --- | --- | --- | --- |
|  | Q1 | Q2 | Q3 | Q4 | Q5 | *P* trend | Per SD increment |
| Lymphocyte percentage, % | ≤ 4.60 | 4.61-6.30 | 6.40-8.30 | 8.40-12.1 | ≥ 12.2 |  |  |
| Deaths/N | 89/166 | 90/164 | 85/163 | 78/168 | 72/167 |  |  |
| crude model | 1 (reference) | 1.16 (0.86-1.55) | 1.00 (0.74-1.35) | 0.86 (0.64-1.17) | 0.77 (0.57-1.05) | 0.0171 | 0.83 (0.74-0.93) |
| model 1 | 1 (reference) | 1.14 (0.85-1.53) | 0.99 (0.74-1.34) | 0.86 (0.63-1.17) | 0.78 (0.57-1.06) | 0.0217 | 0.83 (0.74-0.94) |
| model 2 | 1 (reference) | 1.12 (0.83-1.51) | 0.99 (0.73-1.35) | 0.85 (0.62-1.16) | 0.76 (0.54-1.06) | 0.0257 | 0.81 (0.71-0.92) |
| RDW,fL | ≤ 41.2 | 41.3-42.9 | 43.0-44.4 | 44.5-46.9 | ≥ 47.0 |  |  |
| Deaths/N | 71/164 | 78/160 | 83/173 | 92/158 | 90/173 |  |  |
| crude model | 1 (reference) | 1.24 (0.90-1.71) | 1.23 (0.90-1.69) | 1.61 (1.18-2.19) | 1.40 (1.02-1.91) | 0.0158 | 1.06 (0.97-1.16) |
| model 1 | 1 (reference) | 1.20 (0.87-1.66) | 1.17 (0.85-1.61) | 1.56 (1.11-2.08) | 1.30 (0.95-1.79) | 0.0599 | 1.04 (0.95-1.13) |
| model 2 | 1 (reference) | 1.23 (0.88-1.71) | 1.20 (0.86-1.66) | 1.55 (1.12-2.14) | 1.34(0.97-1.86) | 0.0518 | 1.04 (0.95-1.14) |

Data was represented as numbers and HR (95% CI).

Model 1: adjusted for age (continuous) and sex.

Model 2: adjusted for model 1 plus smoking history, hypertension history, diabetes history, aortic valve replacement history, anatomical classification, etiology, aorta diameter, onset time and hospital centers.
