# Supplementary material for: Association of lymphopenia and RDW elevation with risk of mortality in acute aortic dissection
Source: PLoS One. 2023 Mar 15;18(3):e0283008. doi: 10.1371/journal.pone.0283008 (PMC10016706; doi:10.1371/journal.pone.0283008)
Supplement: Attachment — Submitted filename: accept.docx [file pone.0283008.s008.docx]

Accept
